# Dead or Alive? Identification of Postmortem Blood Through Detection of D-Dimer

**DOI:** 10.3390/biology14070784

**Published:** 2025-06-28

**Authors:** Amy N. Brodeur, Tai-Hua Tsai, Gulnaz T. Javan, Dakota Bell, Christian Stadler, Gabriela Roca, Sara C. Zapico

**Affiliations:** 1 Biomedical Forensic Sciences, Boston University Chobanian & Avedisian School of Medicine, Boston, MA 02118, USA; abrodeur@bu.edu (A.N.B.); thtsai@bu.edu (T.-H.T.); 2 Department of Physical and Forensic Sciences, Alabama State University, Montgomery, AL 36104, USA; gjavan@alasu.edu; 3 Lone Star College Montgomery, Conroe, TX 77384, USA; bell.dakota19@gmail.com; 4 SERATEC^®^ Gesellschaft für Biotechnologie mbH, Ernst-Ruhstrat-Strasse 5, 37079 Göttingen, Germany; stadler@seratec.com (C.S.); gabriela.roca@seratec.com (G.R.); 5 Department of Chemistry and Environmental Science, New Jersey Institute of Technology, Newark, NJ 07102, USA; 6 Anthropology Department and Laboratories of Analytical Biology, National Museum of Natural History, Smithsonian Institution, Washington, DC 20560, USA

**Keywords:** D-dimer, postmortem blood, menstrual blood, immunochromatographic test, DNA profile

## Abstract

At crime scenes, apart from the detection of blood, it may be important to determine whether a person was alive at the time of blood deposition. Based on the rapid onset of fibrinolysis after death, this pathway could be considered to identify potential biomarkers for postmortem blood. Fibrinolysis is the natural process that breaks down blood clots after healing a vascular injury. One of its products, D-dimer, could be a potential biomarker for postmortem blood. SERATEC^®^ (SERATEC^®^ GmbH, Göttingen, Germany) has developed the PMB immunochromatographic assay to simultaneously detect human hemoglobin and D-dimer. The main goals of this study were to assess the possibility of using this test to detect postmortem blood, evaluate D-dimer levels in antemortem, menstrual, and postmortem blood, and assess the ability to obtain STR profiles from postmortem blood. Except for one degraded sample, all postmortem blood samples reacted positively for the presence of D-dimer using the SERATEC^®^ PMB test. All antemortem blood samples from living individuals showed negative results for D-dimer detection, except for one liquid sample with a weak positive result, probably due to pre-existing health conditions. Menstrual blood samples gave variable results for D-dimer. The DIMERTEST^®^ Latex assay was used for semi-quantitative measurement of D-dimer concentrations, with postmortem and menstrual blood yielding higher D-dimer concentrations compared to antemortem blood. Full STR profiles were developed for all postmortem samples tested except for one degraded sample, pointing to the possibility of not only detecting postmortem blood at the crime scene but also the potential identification of the victim.

## Simple Summary

The detection of body fluids at the crime scene is important for the potential identification of the perpetrator and/or the victim. One of the most common fluids is blood. There are different techniques to detect blood at crime scenes; however, it may also be important to determine whether a person was alive at the time of blood deposition. This study uses one of the products of a natural process in our bodies, D-dimer, to evaluate the possibility of identifying blood from a deceased individual. This was carried out by assessing three types of blood—from living people, from deceased people, and menstrual blood—and applying rapid tests like the ones for COVID-19 but detecting D-dimer and a specific assay to quantify the amount of D-dimer. Our results indicated that blood samples from deceased individuals, except for one, were positive in the rapid tests. All living individuals’ blood samples were negative, except for one liquid sample with a weak positive result. Menstrual blood samples gave variable results. Concerning the amount of D-dimer, samples from deceased individuals and menstrual samples presented higher concentrations than living individuals’ blood samples. Blood samples from deceased individuals except one gave full DNA profiles, indicating the possibility of identifying the victim.

## 1. Introduction

Identification of body fluids at the crime scene is of the utmost importance to find biological evidence related to the crime, to identify the perpetrator and/or the victim, and to determine what activities may have taken place at the scene [1]. Research to improve the detection of body fluids has involved existing and novel methods, and includes the measurement of protein catalytic activity [2], microscopic examination [1], and spectroscopic analysis [3,4]. In recent studies, the analysis of microRNA [5], mRNA [6], and DNA methylation profiling [7] has been reported to differentiate bodily fluids successfully.

Blood is one of the most commonly encountered biological samples in criminal investigation. There are two general approaches to identify blood at the crime scene: the classic colorimetric chemical reaction [1] and immunochromatographic tests [8]. The latter are based on antigen-antibody reactions and are known as lateral flow immunochromatographic (LFI) assays. These tests use mobile and stationary monoclonal anti-human antibodies against a specific protein that is unique or highly specific to the target body fluid (i.e., human hemoglobin or glycophorin A in blood), which form a visible pink line in the presence of this protein [6,9]. The aforementioned methods have been validated, and they are currently being used by crime labs and medical examiner’s offices worldwide. Depending on the forensic case, apart from identifying blood, it may also be important to test for postmortem indicators in blood to determine whether the person was alive at the time of blood deposition when a pool of blood is left at the scene. This information may also assist the medical examiner as a factor to consider in estimating the time of death or in determining the primary or secondary crime scene. Thus, identifying blood as postmortem has much potential in forensic science casework.

In order to identify potential biomarkers to detect postmortem blood, it is necessary to focus on the fibrinolysis pathway. Upon the healing of a vascular injury, fibrinolysis is the natural process that prevents a blood clot from growing and causes problems to the body. During the process, the blood clot is lysed through the action of plasmin, an enzyme found in the blood that is generated to break down the covalent crosslinks of the stabilized fibrin polymers [10]. A fibrin thrombus degrades nearly as soon as it is formed. The degradation elevates the level of fibrinogen degradation products (FDPs), which are currently the most widely used indicators of thrombosis [11]. The breakdown of fibrinogen and fibrin produces degradation products of various molecular weights. Among the fragments, the soluble fibrin monomers crosslinked between two adjacent outer D domains, known as D-dimers, are detectable in human plasma. The unique structure of D-dimer enables it to serve as a target to help diagnose and detect deep vein thrombosis (DVT), venous thromboembolism (VTE), pulmonary embolism (PE), and disseminated intravascular coagulation (DIC) [12,13,14]. In contrast, low concentrations of D-dimer are present in normal blood.

Currently, the detection of D-dimer is utilized to presumptively distinguish menstrual blood from peripheral blood in the forensic field. Fibrinolysis plays an important role in menstruation [15]. It is generally accepted that the fluidity of menstrual blood is caused by fibrinolytic activity. Unlike peripheral blood, menstrual blood lacks fibrinogen and fibrin but is rich in FDPs [16]. This feature has been used to distinguish menstrual blood from peripheral blood in cases of alleged sexual assault through an immunochromatographic multiplex assay [17].

Apart from menstrual blood, D-dimer is also elevated in postmortem blood. One study has shown that the concentration of D-dimer in menstrual blood is approximately 200 times greater than in peripheral blood, and the postmortem concentration is two times higher than that in menstrual blood [18]. In the 1980s, research on postmortem fibrinolytic activity was conducted using cadaveric blood [19]. Based on the rapid increase in fibrinolysis after death, it is feasible that the biomarker D-dimer could be incorporated into methods designed to identify the presence of postmortem blood. Rutty et al. (2003) [20] demonstrated high concentrations of D-dimer in postmortem blood, and Sakurada et al. (2005) [21] showed that the latex agglutination assay for D-dimer is valuable in distinguishing between postmortem and antemortem blood in bloodstains [21]. Although the latex agglutination assay is less sensitive than other assays, it provides a rapid and simple test for elevated levels of cross-linked fibrin degradation products in patients with thrombosis (Elms et al., 1986) [22]. Therefore, the latex agglutination assay for D-dimer could be considered as a potential test for distinguishing between postmortem and antemortem blood at crime scenes.

SERATEC^®^ (SERATEC^®^ GmbH, Göttingen, Germany) has developed an immunochromatographic assay called PMB to detect human hemoglobin and D-dimer simultaneously. This test has been primarily developed to identify menstrual blood [17]. However, based on the aforementioned, it is possible that this test could be useful to detect postmortem blood, though there have been only a few attempts to evaluate the feasibility of this test towards this goal [17,23,24].

The aim of this study was to assess the possibility of applying the SERATEC^®^ PMB test to detect postmortem blood, while also evaluating the factors that could affect D-dimer concentration in postmortem samples including the time since death, i.e., postmortem interval (PMI) and anticoagulants added to the collection tubes. A latex agglutination assay was performed to semi-quantitatively assess the levels of D-dimer in peripheral, menstrual, and postmortem blood. Additionally, the ability to obtain STR profiles from postmortem blood was also evaluated in order to potentially identify the decedent.

## 2. Material and Methods

### 2.1. Sample Collection

#### 2.1.1. Postmortem Blood Samples

Forty-one postmortem blood samples were collected during autopsy and stored frozen until use. Multiple agencies provided samples; thus, the collection technique and information provided varied. The site of collection within the body and reported cause of death was unknown for most samples, though sex, age, and approximate time between death and sample collection were noted (Table S1). The New Jersey Institute of Technology Institutional Review Board (IRB) committee approved all the procedures related to body fluid experimentation (protocol number: 2110013076).

#### 2.1.2. Antemortem Peripheral Blood Samples

Fourteen antemortem peripheral blood samples were collected in accordance with a protocol (H-26187) approved by Boston University IRB, and seven were purchased from BiolVT^®^ (New York, NY, USA). All peripheral bloodstain samples were stored frozen upon receipt.

#### 2.1.3. Menstrual Blood Samples

Eighteen menstrual bloodstain samples were collected from thirteen volunteers (IRB protocol H-26187) and stored frozen upon receipt. For five donors, samples from two separate days during one menstrual period were collected. The menstrual samples were collected in two ways: in liquid form or using a cotton swab that was subsequently air dried. No preservatives or anticoagulants were added to the samples.

### 2.2. Immunochromatographic Assay

The SERATEC^®^ PMB test (SERATEC^®^ GmbH, Göttingen, Germany) simultaneously detects human hemoglobin and D-dimer. It is reported to have a limit of detection of 20 ng/mL for human hemoglobin and 400 ng/mL for D-dimer [25]. For negative results, only one line (the C, control line) is visible, which demonstrates that the test is functioning properly. For positive results, three pink lines are visible at the result window after 10 min (C, control; M, D-dimer; P, peripheral blood), indicating the detection of human hemoglobin and D-dimer. If only two lines at C and P are visible, only human hemoglobin is detected; thus, only peripheral blood is present. Examples of strong, weak, and negative results are presented in Supplementary Figure S1. A weak positive result was designated as clearly visible but substantially lighter than the band appearing in positive results. Two different sample preparations were carried out to perform the test: liquid samples and bloodstain samples.

#### 2.2.1. Liquid Samples

All liquid blood samples were diluted 1:100 before testing by adding 1.5 µL of the blood sample to 150 µL of the SERATEC^®^ extraction buffer provided. After 10 min, three drops of the extracted solution (approximately 120 µL) were added to the sample well using the dropper provided. Results were read after 10 min at room temperature.

#### 2.2.2. Bloodstain Samples

Bloodstains were made by applying 100 µL of liquid blood onto filter paper or two drops of blood onto a cotton fabric swatch. The stains were allowed to dry for one day and stored frozen until use. Cuttings of the stains measuring approximately 1 cm^2^ were extracted for up to 60 min by adding them directly into the vial containing 1.5 mL of extraction buffer. After the extraction was completed, three drops of the extracted solution (approximately 120 µL) were added to the sample well using the dropper provided. Results were read after 10 min at room temperature.

### 2.3. Rapid Latex Agglutination Assay

The DIMERTEST^®^ Latex assay (Siemens Healthineers, Erlangen, Germany) was used to semi-quantitatively measure cross-linked fibrin degradation products using a highly specific D-dimer monoclonal antibody coated with latex beads. When the D-dimer concentration is above 200 ng/mL, the D-dimer fragments in blood bind to the coated latex beads, causing visible agglutination to occur.

#### 2.3.1. Sample Preparation

Two-fold serial dilutions (1:2 through 1:256) of liquid blood samples were made with the buffer provided with the kit. All reagents were brought to room temperature before testing.

#### 2.3.2. Test Procedure

One drop of the latex reagent was placed within a well on the test card by holding the reagent bottle vertically without touching the surface of the test card. Then, 20 μL of the undiluted blood sample or one drop of the control solutions provided was added in the same well beside the drop of the latex reagent using a pipette. The sample and reagent were mixed with the stirrer provided until uniformly distributed and the test card was rocked gently by hand. Results were read at exactly three minutes under a strong light source. To ensure the proper function of the test, positive and negative controls were performed in each batch of tests. The observation of visible agglutination indicated a positive result for D-dimer, whereas the absence of agglutination indicated a negative result with a D-dimer level of less than 200 ng/mL. Positive results were compared against values provided by the manufacturer, where D-dimer concentration ranges corresponded to the largest dilutions for which visible agglutination was observed.

### 2.4. DNA Extraction

Total DNA was isolated from cotton swabs of some of the postmortem samples using a modification of the DNeasy Blood and Tissue Kit (Qiagen^®^, Hilden, Germany) protocol [26]. The swab was diluted in 400 µL SERATEC^®^ extraction buffer. Then, the swab was kept in a microcentrifuge tube, and 20 µL of Proteinase K and 400 µL of Reagent AL were added. The mixture was vortexed for 15 s and incubated under agitation at 56 °C for 10 min. After that, 400 µL of ethanol was added and vortexed for 15 s. The mixture was transferred to the column following the manufacturer’s protocol. DNA was recovered in 50 µL of AE buffer.

### 2.5. DNA Quantification

Human-DNA-specific quantification was carried out using the PowerQuant System (Promega Corporation, Madison, WI, USA) along with the QuantStudio (ThermoFisher Scientific, Waltham, MA, USA) according to the manufacturer’s protocol. This included a positive control, with known DNA concentration; and two negative controls, with all the reaction components and water instead of the DNA template.

### 2.6. Nuclear DNA Profiling

The Promega PowerPlex^®^ Fusion 6C System (Promega Corporation, Madison, WI, USA) was used to amplify and characterize 23 autosomal STRs, 3 Y-STRs, and Amelogenin in 15 µL of DNA, according to the manufacturer’s protocol, including a negative control (water) and a positive control (2800M Control DNA). Fragment analysis was carried out on SeqStudio (ThermoFisher Scientific, Waltham, MA, USA) under the following parameters: 7 s injection time; 1200 V injection voltage; 1440 s run time; 9000 V run voltage. DNA profiling was determined through Microsatellite Analysis software available on the Thermo Fisher Cloud (ThermoFisher Scientific, Waltham, MA, USA), with an analytical threshold of 150 RFU.

## 3. Results

### 3.1. SERATEC^®^ PMB Test

#### 3.1.1. Postmortem Liquid Blood Samples

Twenty-one postmortem liquid blood samples (P021-P041) were tested with the SERATEC^®^ PMB test. All samples showed positive results for both hemoglobin and D-dimer except for one with no added anticoagulant, which had a negative result for D-dimer. The poor condition of the sample, which included precipitations of particulate matter and a rotten odor, was suspected of causing the negative result.

#### 3.1.2. Antemortem Liquid Blood Samples

Eleven liquid antemortem peripheral blood samples were used for this test. All antemortem samples were positive for hemoglobin when tested using the SERATEC^®^ PMB test. One of the antemortem samples produced a weak positive result for D-dimer, whereas all of the other antemortem samples were negative (Table 1).

#### 3.1.3. Menstrual Liquid Blood Samples

All four of the menstrual blood samples acquired and tested in liquid form tested positive for hemoglobin and D-dimer using the SERATEC^®^ PMB test.

#### 3.1.4. Postmortem Bloodstain Samples

All of the postmortem bloodstain samples reacted positively for the presence of both hemoglobin and D-dimer (Table 2). For six of the cassettes, the hemoglobin line was very faint and difficult to visualize. The high-dose hook effect was suspected in these samples; therefore, a dilution process was performed, and testing was repeated. Upon retesting, the six diluted postmortem blood samples reacted positively for both hemoglobin and D-dimer, displaying pink lines that were readily visible (Table 3). Hence, the positive rate for D-dimer presence in postmortem bloodstain samples tested using the SERATEC^®^ PMB test was 100%. The band intensity in both hemoglobin detection and D-dimer detection varied among samples.

#### 3.1.5. Antemortem Bloodstain Samples

Ten antemortem peripheral blood samples were collected directly onto a clean substrate and allowed to air dry. The types of substrates included cotton swatches, filter paper, and gauze pads. All substrates were white in color, and no other contaminants or preservatives were present.

All antemortem peripheral bloodstain samples reacted positively to the presence of hemoglobin, while none reacted positively to the presence of D-dimer (Table S2). Nine peripheral bloodstain samples presented strong positive signals, and one sample gave a moderate positive signal for hemoglobin. No additional dilution was required for peripheral bloodstain samples since no high-dose hook effect, other than perhaps the one sample with a moderate signal, was observed among antemortem peripheral bloodstain samples.

#### 3.1.6. Menstrual Bloodstain Samples

These menstrual blood samples were collected in the form of bloodstains on a white panty liner or cotton swatch. All eleven menstrual bloodstain samples tested reacted positively for the presence of hemoglobin. Nine menstrual bloodstain samples also reacted positively for D-dimer (Table 4). One menstrual bloodstain sample that initially showed very faint bands (M001-Day3) for hemoglobin detection due to a suspected high-dose hook effect was diluted, and when retested, the results displayed positive signals for both hemoglobin and D-dimer presence, although only one of the triplicate samples demonstrated an increase in band intensity. The two samples with no detectable D-dimer were from the same donor (M002) on two consecutive days of menstruation.

### 3.2. DIMERTEST^®^ Latex Assay

Serial dilutions of liquid antemortem, postmortem, and menstrual blood samples starting from 1:2 and subsequently reducing each by a factor of two were prepared and tested for semi-quantitative purposes. The approximate range of the D-dimer level was estimated based on the table provided by the kit. The D-dimer concentration reported for each blood sample is based on to the largest dilutions for which visible agglutination was observed. The largest dilution tested was 1:256, corresponding to a concentration range of 51.2–102.4 mg/L.

**Figure 1:**
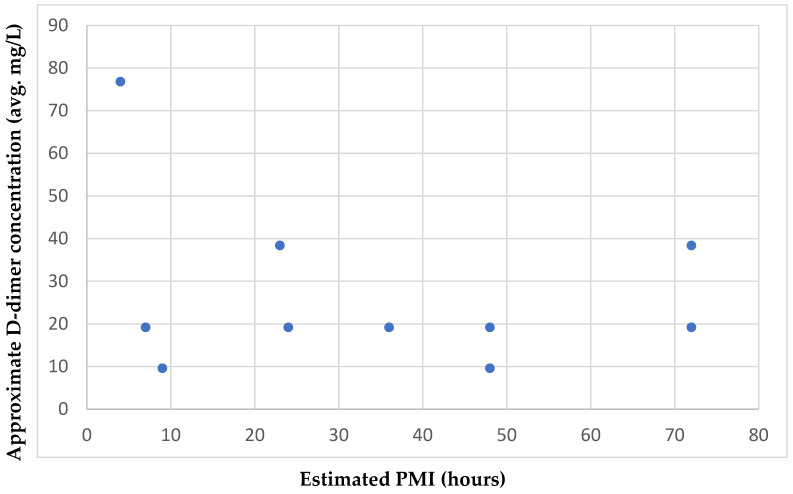
Approximate D-dimer concentration and estimated PMIs of the postmortem blood samples. The average D-dimer concentration in ten postmortem blood samples and corresponding postmortem interval is displayed.

Seven of the nine antemortem samples tested showed a negative result, indicating a D-dimer level lower than 0.2 mg/L. The antemortem sample with the highest concentration range corresponded to 0.8–1.6 mg/L. The ten postmortem samples had concentrations in the ranges of 6.4–12.8 mg/L and 51.2–102.4 mg/L. The five menstrual samples showed D-dimer levels in the ranges of 12.8–25.6 mg/L and 51.2–102.4 mg/L (Table 5). Comparison of the estimated PMIs for the ten postmortem samples analyzed and their corresponding approximate D-dimer concentration ranges showed no relationship between the increase in PMI and the D-dimer concentration in the samples, based on their distribution on the scatter plot (Figure 1).

**Figure 2:**
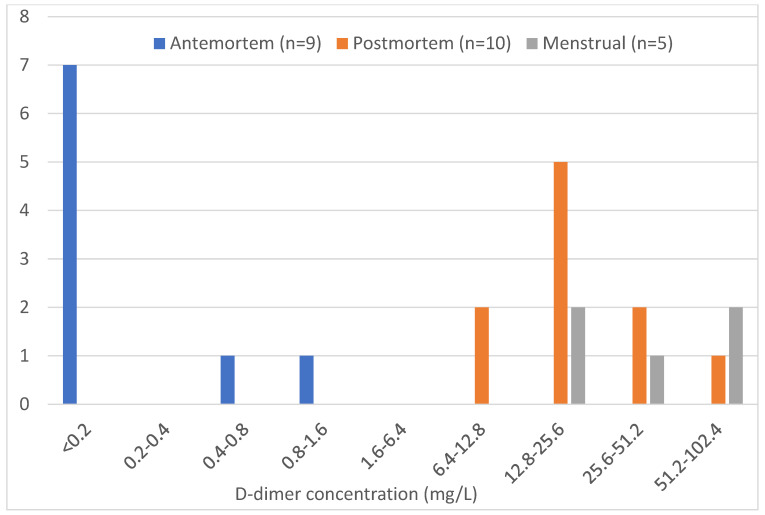
Sample counts of each blood type according to the approximate concentration of D-dimer. The antemortem, postmortem, and menstrual blood sample counts are shown based on the approximate ranges of D-dimer levels detected using the DIMERTEST^®^ Latex assay.

The antemortem samples were primarily negative for D-dimer, indicating a concentration below the threshold for this assay. The highest concentration range of D-dimer detected for the antemortem samples was lower than the lowest concentration ranges of the postmortem and menstrual blood samples, indicating that the concentration of D-dimer in the antemortem samples was substantially lower than in the postmortem and menstrual blood samples, as expected. Postmortem samples had a wider concentration range than the other two sample types, with most samples in the 12.8–25.6 mg/L range. The highest concentration range in menstrual blood samples was the same as in postmortem samples, indicating no real difference in D-dimer concentrations between the two types (Figure 2).

### 3.3. DNA Extraction and Quantification

Fourteen postmortem samples were subjected to DNA extraction. Human-specific DNA quantitation was carried out with the PowerQuant system Software Version: 4.8.0.0 (Promega Corporation, Madison, WI, USA), obtaining quantifiable DNA in all of the samples, except in P032, the one postmortem blood sample that was negative for D-dimer. The degradation ratios were variable among samples, but the IPC shift, to detect inhibitors, was below the threshold in all samples (Table 6).

### 3.4. STR Profiling

DNA profiling was performed on samples with quantifiable DNA using between 0.5 and 1 ng of DNA, according to the manufacturer’s instructions. All samples yielded full, good-quality DNA profiles, independent of the time since death (Figure S2).

## 4. Discussion

This study explored the identification of postmortem blood through the detection of D-dimer. No antemortem donor medical information was known for any of the blood samples tested.

All postmortem male and female samples (liquid blood and bloodstains) reacted positively for the presence of hemoglobin using the SERATEC^®^ PMB tests, and all of them except one putrefied sample showed a positive result for D-dimer detection. The positive reaction rate of hemoglobin and D-dimer is consistent with previous studies [17,23]. The study by Holtkötter et al. demonstrated a positive reaction rate for the D-dimer in 70% of the postmortem samples (7 out of 10). A recent study by Konrad et al. [23] also detected D-dimer in postmortem samples applying the same test. Additionally, previous research has shown that menstrual blood and postmortem blood could not be distinguished by FDP detection as both contain large amounts of FDP; thus, the high positive result rate for D-dimer among postmortem blood samples was expected [18]. This points to the potential use of D-dimer as an indicator for identifying postmortem blood.

During autopsy or body preservation, postmortem blood samples are generally taken from the deceased’s heart, the thoracic cavity, or the jugular vein. The FDP level has been reported to be higher in the heart than in the thoracic cavity in cadavers [19]. It is known that the level of D-dimer and the prevalence of elevated D-dimer increases with age, so the origin of FDP detected from elderly donors should be considered, as the detected FDP could be either from antemortem fibrinolysis or from postmortem fibrinolysis [24]. D-dimer was detectable among the postmortem samples, regardless of the age of the donor.

Another factor to consider is whether an anticoagulant was added to the blood tubes at the time of collection, prior to testing. The only postmortem sample that tested negative for D-dimer was a foul-smelling sample with no preservatives or anticoagulants added (P032). In an attempt to detect reduced levels of D-dimer, the test development duration was extended to 60 min; however, the results remained negative. Additionally, the condition of the sample, containing solid particles and a foul odor, impacted the success of DNA analysis. When flesh and tissue decay, decarboxylation of amino acids produces a rotten odor [27]. Although an approximate PMI of 22 h was given, information on the body’s physical condition before blood collection, and environmental factors such as the temperature and humidity of the location where the body was found, were not provided. Two other postmortem samples with no added preservatives gave positive results for D-dimer, as did all of the samples containing EDTA, sodium heparin, and sodium fluoride/potassium oxalate. Thus, the type of preservative added did not appear to have an impact on D-dimer detection.

D-dimer levels in blood from different postmortem intervals have been assessed here with no obvious correlation observed. Of twenty postmortem bloodstains (PMI = 4–91 h) tested with the SERATEC^®^ PMB test, all but two gave strong positive results for D-dimer. The samples displaying moderately strong positive results had PMIs of 72 h and 9 h. One study that investigated the relationship between FDP and time after death in rats reported that FDP increased rapidly and reached a maximum value 12 to 20 h after death in all rats, regardless of the cause of death [19]. While that study did not investigate FDP levels beyond one day postmortem, D-dimer was detectable via immunochromatographic assay up to four days after death in the current study.

All peripheral liquid and bloodstain samples from living individuals showed positive results for hemoglobin detection, and negative results for D-dimer detection, except for one liquid blood sample (A005), giving a weak positive result. No information on the donor’s medical history was provided; therefore, the possibility of D-dimer elevation caused by health factors could not be excluded. In healthy adults, the D-dimer concentration in liquid peripheral blood is about 47 ng/mL, and in peripheral bloodstains, the D-dimer concentration is about 11 ng/mL [18]. Thus, it should be below the threshold of the SERATEC^®^ PMB test (400 ng/mL). Despite this weak positive result, this sample was tested for D-dimer using the DIMERTEST^®^ Latex assay, giving negative results, indicating that the detected D-dimer concentration was less than 0.2 mg/L.

With respect to menstrual blood samples, although all of them (liquid and bloodstains) gave positive results for hemoglobin, there was variability regarding the detection of D-dimer. Notably, the two samples that showed no detectable levels of D-dimer using the SERATEC^®^ PMB test were from the same donor (M002). Further studies will be conducted to better understand this observation and investigate potential influencing factors. As menstrual blood evaluation was not the main objective of this work, pre-existing samples in the laboratory were used that were self-collected under varying conditions. For some of the samples, the day of the menstrual cycle on which the sample was collected was noted; for others, it was not. When this information was provided, there seemed to be variation in D-dimer levels within a single individual at different time points. Thus, there could be intrinsic factors impacting the detection of D-dimer in menstrual blood. Some samples that were previously analyzed (and showed no detectable D-dimer after 10 min using the SERATEC^®^ PMB test were retested, increasing the reading time up to 60 min. About half of these samples had a positive reaction following extended development duration (Table S3). In this situation, no reliable conclusions can be drawn about the detectability of D-dimer within this sample set. The study of D-dimer activity over time will be approached in another work. 

Immunoassay cards are not only used in the forensic field but also for screening for medical conditions, like infectious diseases [28]. According to a study evaluating the time needed for positive results in one COVID-19 antigen test, the duration required to obtain positive results was inversely proportional to the quantity of virus the sample contained [29]. Similarly, the results in the present study have shown that for suspected low-concentration samples, extended reading time increases the sensitivity of the SERATEC^®^ PMB test.

The DIMERTEST^®^ Latex assay was used for semi-quantitative measurement of D-dimer concentrations. Of the nine antemortem samples, two showed a positive reaction between the concentrations of 0.4–0.8 mg/L and 0.8–1.6 mg/L. In contrast, both samples tested negative for D-dimer using the SERATEC^®^ PMB test, which has a cut-off value of 400 ng/mL (0.4 mg/L).

In the study of Sakurada et al., D-dimer levels in nine postmortem blood postmortem blood samples ranged from 335 to 2800 µg/mL (mg/L), and four antemortem blood samples were negative (<0.52 µg/mL) when tested using a latex agglutination assay, which is generally consistent with the results of the antemortem blood samples in this research [21]. However, in this research, the D-dimer concentration range of the postmortem blood samples was lower than Sakurada had reported, which was approximately 6.4–102.4 mg/L (µg/mL). The discrepancy may be because Sakurada used a latex photometric immunoassay system for quantification. Given that the DIMERTEST^®^ Latex assay was used semi-quantitatively and the agglutination was read subjectively by eye, the concentration range assigned may not be as accurate.

In Miyaishi’s study, the mean plasma concentration of D-dimer in menstrual blood was reported to be 102 µg/mL (mg/L) [18], which falls in the approximate concentration range measured in the menstrual blood samples (12.8–102.4 mg/L) in this research. The mean plasma concentration of D-dimer in postmortem blood samples was higher than in menstrual blood samples in Miyaishi’s study, whereas the D-dimer concentration range measured in the postmortem blood (12.8–102.4 mg/L) was the same as menstrual blood samples (12.8–102.4 mg/L) for this research.

In Sakurada’s study [21], the relationship between the time of death and the D-dimer level was not discussed due to the difficulty in sample collection, so no comparisons between PMI and D-dimer concentration can be made. This research found no correlation between the PMI and D-dimer concentration in the postmortem blood samples. D-dimer elevation is known to have multiple causes, and it is probable that other factors, besides PMI duration, contributed to the increase in D-dimer level in postmortem blood.

Overall, most of the antemortem blood samples were negative for D-dimer, indicating they had a D-dimer concentration lower than the cut-off value for the assay. The lowest approximate concentration of D-dimer in postmortem blood samples was four times higher, and in menstrual blood samples 8 times higher, than the highest concentration in antemortem samples. This research demonstrated that compared to antemortem blood samples, postmortem and menstrual blood samples had similar, elevated D-dimer concentrations.

Regarding DNA extraction and profiling from postmortem blood, all samples tested except one (P032) yielded quantifiable DNA with variability in the degradation ratio, but without inhibitors; the degradation ratios were not related to the PMI. This variability was pointed out in the early years of DNA analysis [30], and also in later studies [31,32]. In this study, except for the putrid sample without preservatives and despite the degradation index, all postmortem samples showed full, good-quality DNA profiles, being able to identify the decedent, if needed, depending on the case.

## 5. Conclusions

This study is one of the few assessing the potential applicability of D-dimer to detect postmortem blood. The SERATEC^®^ PMB immunochromatographic test efficiently detected D-dimer in all postmortem blood samples except for a single degraded one. Only 1 of 21 antemortem peripheral blood samples was weakly positive for D-dimer, and D-dimer band intensity in menstrual blood samples was variable, with one donor having no detectable D-dimer. D-dimer concentrations were higher in postmortem and menstrual blood and almost undetectable in antemortem blood using the semi-quantitative DIMERTEST^®^ Latex assay. It was possible to obtain good-quality STR profiles from postmortem blood samples, irrespective of PMI.

Detection of D-dimer in postmortem blood samples did not appear to be impacted by variations in sex, age, time since death, collection site, cause of death, or blood preservative used. However, a limitation of this study is that pertinent information was not complete for all samples. In particular, the exact postmortem interval, perimortem environmental conditions, medical history, and anatomical site of blood collection were unknown for many samples, limiting the veracity of conclusions that can be drawn about D-dimer levels in these samples. Additionally, it should be considered, based on the results, that this test may not be able to distinguish between menstrual and postmortem blood at crime scenes and antemortem blood from an injury sustained by a person with a previous medical history of DVT, VTE, PE, or DIC.

Future research will expand the menstrual blood results, including a variety of samples to test the effectiveness of D-dimer as a marker for detecting this type of blood.

## Figures and Tables

**Table 1:** SERATEC^®^ PMB test results of liquid antemortem blood samples. The biological sex, anticoagulant added in the collection tube, and results for hemoglobin and D-dimer tested using the SERATEC^®^ PMB test are shown for each sample. A weak positive result was designated as clearly visible but substantially lighter than the band appearing in positive results.

Antemortem Sample	Gender	Anticoagulant in the Collection Tube	SERATEC^®^ PMB Test	
			D-Dimer	Hemoglobin
**A011**	F	None	Negative	Positive
**A012**	F	None	Negative	Positive
**A013**	F	EDTA	Negative	Positive
**A014**	M	EDTA	Negative	Positive
**A015**	F	EDTA	weak positive	Positive
**A016**	M	EDTA	Negative	Positive
**A017**	Pooled	EDTA	Negative	Positive
**A018**	Pooled	EDTA	Negative	Positive
**A019**	Pooled	EDTA	Negative	Positive
**A020**	Pooled	EDTA	Negative	Positive
**A021**	Pooled	EDTA	Negative	Positive

**Table 2:** SERATEC^®^ PMB test results of postmortem bloodstain samples. The results of D-dimer detection are shaded in the table according to intensity. Each sample was tested in triplicate, and most samples showed consistent results among triplicates.

Postmortem Sample	PMB Test					
	Trial 1		Trial 2		Trial 3	
	Hemoglobin	D-Dimer	Hemoglobin	D-Dimer	Hemoglobin	D-Dimer
**P001**	Strong+	Strong+	Strong+	Strong+	Strong+	Strong+
**P002**	Weak+	Strong+	Weak+	Strong+	Weak+	Strong+
**P003**	Strong+	Strong+	Strong+	Strong+	Strong+	Strong+
**P004**	Weak+	Strong+	Weak+	Strong+	Weak+	Strong+
**P005**	Strong+	Strong+	Strong+	Strong+	Strong+	Strong+
**P006**	Moderate+	Moderate+	Moderate+	Moderate+	Moderate+	Moderate+
**P007**	Strong+	Strong+	Strong+	Strong+	Strong+	Strong+
**P008**	Weak+	Strong+	Weak+	Strong+	Weak+	Strong+
**P009**	Weak+	Strong+	Weak+	Strong+	Weak+	Strong+
**P010**	Strong+	Strong+	Strong+	Strong+	Strong+	Strong+
**P011**	Strong+	Strong+	Strong+	Strong+	Strong+	Strong+
**P012**	Weak+	Strong+	Weak+	Strong+	Weak+	Strong+
**P013**	Weak+	Strong+	Weak+	Strong+	Weak+	Strong+
**P014**	Strong+	Strong+	Strong+	Strong+	Strong+	Strong+
**P015**	Weak+	Strong+	Weak+	Strong+	Weak+	Strong+
**P016**	Moderate+	Strong+	Strong+	Strong+	Moderate+	Strong+
**P017**	Moderate+	Moderate+	Moderate+	Moderate+	Moderate+	Moderate+
**P018**	Strong+	Strong+	Strong+	Strong+	Strong+	Strong+
**P019**	Strong+	Strong+	Strong+	Strong+	Strong+	Strong+
**P020**	Moderate+	Strong+	Moderate+	Strong+	Moderate+	Strong+

**Table 3:** SERATEC^®^ PMB test results of diluted postmortem bloodstain samples. The results of D-dimer detection are shaded in the table according to intensity. Initially, these samples showed weak positive bands for hemoglobin. Subsequent to dilution, all samples showed strong or moderate intensity for both hemoglobin and D-dimer detection. Each sample was tested in triplicate, and most samples showed consistent results among triplicates.

Postmortem Sample	PMB Test					
	Trial 1		Trial 2		Trial 3	
	Hemoglobin	D-Dimer	Hemoglobin	D-Dimer	Hemoglobin	D-Dimer
**P002-Dil**	Strong+	Strong+	Moderate+	Strong+	Strong+	Strong+
**P004-Dil**	Moderate+	Strong+	Moderate+	Strong+	Moderate+	Strong+
**P008-Dil**	Strong+	Strong+	Strong+	Strong+	Strong+	Strong+
**P009-Dil**	Moderate+	Strong+	Moderate+	Strong+	Moderate+	Strong+
**P012-Dil**	Moderate+	Moderate+	Moderate+	Strong+	Moderate+	Strong+
**P013-Dil**	Moderate+	Moderate+	Moderate+	Moderate+	Moderate+	Moderate+

**Table 4:** SERATEC^®^ PMB test results of menstrual bloodstain samples. The results of D-dimer detection are shaded according to intensity. Dilution was performed when the intensity of hemoglobin results was weak, possibly due to a high-dose hook effect. Each sample was tested in triplicate, and most samples showed consistent results among triplicates.

Menstrual Blood Sample	PMB Test					
	Trial 1		Trial 2		Trial 3	
	Hemoglobin	D-Dimer	Hemoglobin	D-Dimer	Hemoglobin	D-Dimer
**M001-Day3**	Weak+	Weak+	Weak+	Weak+	Weak+	Weak+
**M001-Day3-Diluted**	Weak+	Weak+	Weak+	Weak+	Moderate+	Moderate+
**M001-Day4**	Moderate+	Weak+	Moderate+	Weak+	Moderate+	Weak+
**M002-Day2**	Strong+	Negative	Strong+	Negative	Strong+	Negative
**M002-Day3**	Strong+	Negative	Strong+	Negative	Strong+	Negative
**M003-Day2**	Strong+	Weak+	Strong+	Weak+	Strong+	Weak+
**M003-Day3**	Strong+	Weak+	Strong+	Weak+	Strong+	Negative
**M004-Day2**	Strong+	Weak+	Strong+	Weak+	Strong+	Weak+
**M004-Day3**	Moderate+	Moderate+	Moderate+	Moderate+	Moderate+	Moderate+
**M005-Day1**	Strong+	Moderate+	Strong+	Moderate+	Strong+	Moderate+
**M005-Day3**	Strong+	Strong+	Strong+	Strong+	Strong+	Strong+

**Table 5:** Approximate range of D-dimer level tested with DIMERTEST^®^ Latex assay. The approximate range of D-dimer levels in nine antemortem, ten postmortem, and five menstrual blood samples with DIMERTEST^®^ Latex assay are listed.

Antemortem Sample	Approx. D-Dimer (mg/L)	Postmortem Sample	Approx. D-Dimer (mg/L)	Menstrual Sample	Approx. D-Dimer (mg/L)
**A001**	0.8–1.6	**P018**	51.2–102.4	**M001**	51.2–102.4
**A002**	0.4–0.8	**P037**	12.8–25.6	**M002**	12.8–25.6
**A003**	<0.2	**P029**	25.6–51.2	**M008**	51.2–102.4
**A004**	<0.2	**P024**	12.8–25.6	**M003**	25.6–51.2
**A005**	<0.2	**P028**	12.8–25.6	**M004**	12.8–25.6
**A006**	<0.2	**P017**	6.4–12.8		
**A007**	<0.2	**P004**	12.8–25.6		
**A009**	<0.2	**P040**	12.8–25.6		
**A010**	<0.2	**P026**	6.4–12.8		
		**P010**	25.6–51.2		

**Table 6:** Human-specific DNA quantitation of fourteen postmortem samples. The DNA concentration (ng/µL), degradation ratio, and IPC shift are shown.

Sample	DNA Concentration (ng/µL)	Degradation Ratio	IPC Shift
P001	12.0279	0.98	0.05
P002	0.2939	1.61	−0.14
P003	6.9269	7.2	0.02
P004	2.1811	11.78	−0.25
P005	0.2146	11.73	−0.62
P006	0.0674	3.72	−0.57
P007	1.3605	2.21	−0.21
P008	0.8472	1.4	−0.26
P009	1.2457	2.34	−0.02
P010	11.9166	2.28	0.01
P022	0.0485	0	−0.10
P029	0.0873	1.26	−0.19
P032	Undetermined	Undetermined	−0.4
P037	2.1875	1.34	−0.37

## Data Availability

Data available upon request.

## Supplementary Materials

The following supporting information can be downloaded at https://www.mdpi.com/article/10.3390/biology14070784/s1: Table S1. Postmortem blood samples; Table S2. SERATEC^®^ PMB test results of antemortem peripheral bloodstain samples; Table S3. SERATEC^®^ PMB test results of blood samples retested for 60 min; Figure S1. Examples of strong (a), weak (b), and negative (c) results; Figure S2. Full DNA profile from Postmortem Sample P001.
