# Supplementary material for: Dead or Alive? Identification of Postmortem Blood Through Detection of D-Dimer
Source: Biology (Basel). 2025 Jun 28;14(7):784. doi: 10.3390/biology14070784 (PMC12292195; doi:10.3390/biology14070784)
Supplement: Supplementary file 1 [file biology-14-00784-s001.zip › biology-3707891-supplementary.pdf]

Supplementary Material

Table S1. Postmortem Blood Samples. Information about postmortem blood sample donors including biological sex, age, approximate PMI, and site of blood collection. N/A, not available.

Postmortem Sample	Biological Sex	Age	Site of Collection	Anticoagulant in Collection Tube	PMI (approx.)	Additional information provided
**P001**	F	N/A	N/A	N/A	N/A	N/A
**P002**	M	60	Cardiac	NaF/KOx	24 hrs	Charring on >90% of body; no thromboemboli
**P003**	M	44	Cavity	NaF/KOx	24 hrs	No pulmonary embolism
**P004**	M	58	Cavity	NaF/KOx	24 hrs	N/A
**P005**	M	75	N/A	NaF/KOx	24 hrs	3rd and 4th degree burns on 100% of body; no thromboembolism in pulmonary arteries
**P006**	M	66	Heart	NaF/KOx	72 hrs	No known thrombus
**P007**	M	75	N/A	NaF/KOx	43 hrs	No thrombi in atria or ventricles
**P008**	M	59	Cardiac	NaF/KOx	22 hrs	No pulmonary emboli
**P009**	F	58	Chest	NaF/KOx	24 hrs	N/A
**P010**	F	58	Chest	NaF/KOx	72 hrs	N/A
**P011**	F	58	N/A	NaF/KOx	24 hrs	N/A
**P012**	F	52	N/A	NaF/KOx	91 hrs	No pulmonary thromboemboli; multiple pulmonary contusions
**P013**	M	54	N/A	NaF/KOx	38 hrs	no emboli in pulmonary arteries
**P014**	M	58	N/A	NaF/KOx	24 hrs	No thromboemboli
**P015**	M	21	N/A	NaF/KOx	18 hrs	Normal pulmonary arteries
**P016**	M	60	Cardiac	NaF/KOx	48 hrs	Thermal flame burns with areas of charring; no thromboemboli in pulmonary arteries
**P017**	F	83	Jugular vein	EDTA	9 hrs	N/A
**P018**	F	90	Jugular vein	EDTA	4 hrs	N/A

**P019**	M	30	N/A	Sodium heparin	44 hrs	No vascular disease
**P020**	M	52	N/A	Sodium heparin	46 hrs	No vascular disease
**P021**	F	33	left femoral vein, descending thoracic aorta, right subclavian	EDTA	14 hrs	N/A
**P022**	F	53	N/A	EDTA	48 hrs	Drowning
**P023**	M	30	N/A	EDTA	19 hrs	GSW to head
**P024**	M	40	N/A	EDTA	48 hrs	Hanging
**P025**	M	34	N/A	EDTA	17 hrs	Multiple drug intoxication
**P026**	F	31	N/A	NaF/KOx	48 hrs	Multiple drug intoxication
**P027**	M	47	N/A	EDTA	12 hrs	Multiple drug intoxication
**P028**	M	31	N/A	None	72 hrs	Multiple drug intoxication
**P029**	M	58	N/A	EDTA	23 hrs	Shotgun to head
**P030**	M	56	N/A	EDTA	16 hrs	Drowning
**P031**	M	43	N/A	EDTA	16 hrs	Hanging
**P032**	M	17	N/A	None	22 hrs	Blunt force
**P033**	F	50	N/A	EDTA	46 hrs	Heart disorder
**P034**	M	70	N/A	EDTA	48 hrs	Multiple drug intoxication
**P035**	M	51	N/A	EDTA	56 hrs	Pneumonia
**P036**	M	21	N/A	EDTA	12 hrs	Mechanical asphyxia
**P037**	F	29	N/A	EDTA	7 hrs	Acute MI/coronary thrombosis
**P038**	M	24	N/A	EDTA	14 hrs	GSW to head
**P039**	F	45	N/A	EDTA	48 hrs	Multiple drug intoxication
**P040**	M	30	N/A	EDTA	36 hrs	Terminal seizure
**P041**	M	37	N/A	None	14 hrs	Multiple drug intoxication

Table S2. SERATEC® PMB Test results of antemortem peripheral bloodstain samples. All samples showed a negative result in D-dimer detection and 90% of those samples showed a strong intensity in hemoglobin detection. No further dilution was needed for any sample in this group.

Antemortem Peripheral Blood Sample	PMB Test					
	Trial 1		Trial 2		Trial 3	
	Hemoglobin	D-Dimer	Hemoglobin	D-Dimer	Hemoglobin	D-Dimer
A001	Moderate +	Negative	Moderate +	Negative	Moderate +	Negative
A002	Strong +	Negative	Strong +	Negative	Strong +	Negative
A003	Strong +	Negative	Strong +	Negative	Strong +	Negative
A004	Strong +	Negative	Strong +	Negative	Strong +	Negative
A005	Strong +	Negative	Strong +	Negative	Strong +	Negative
A006	Strong +	Negative	Strong +	Negative	Strong +	Negative
A007	Strong +	Negative	Strong +	Negative	Strong +	Negative
A008	Strong +	Negative	Strong +	Negative	Strong +	Negative
A009	Strong +	Negative	Strong +	Negative	Strong +	Negative
A010	Strong +	Negative	Strong +	Negative	Strong +	Negative

Table S3. SERATEC® PMB Test results of blood samples retested for 60 minutes. The sample type and the results for hemoglobin (Hb) and D-dimer observed at 10-minute increments are shown. A weak positive (+) was designated as clearly visible but substantially lighter than the band appearing in positive results. A very weak positive was designated as visible but faint compared to the band appearing in weak positive results.

Sample	Sample type	SERATEC® PMB Test						
		Hb	D-dimer					
			10 min	20 min	30 min	40 min	50 min	60 min
Peripheral blood (n=4)	antemortem	+	Negative	Negative	Negative	Negative	Negative	Negative
M010	menstrual	+	Negative	Negative	Very weak +	Weak +	Weak +	Weak +
M012	menstrual	+	Negative	Negative	Very weak +	Very weak +	Very weak +	Very weak +
M013	menstrual	+	Negative	Negative	Negative	Negative	Negative	Negative
M002-Day2	menstrual	+	Negative	Negative	Very weak +	Weak +	Weak +	Weak +

**M002-Day3**	menstrual	+	Negative	Negative	Negative	Negative	Negative	Negative
**M014**	menstrual	+	Negative	Negative	Negative	Negative	Negative	Negative
**P022**	postmortem	+	Negative	Negative	Negative	Very weak +	Very weak +	Very weak +
**P032**	postmortem	+	Negative	Negative	Negative	Negative	Negative	Negative

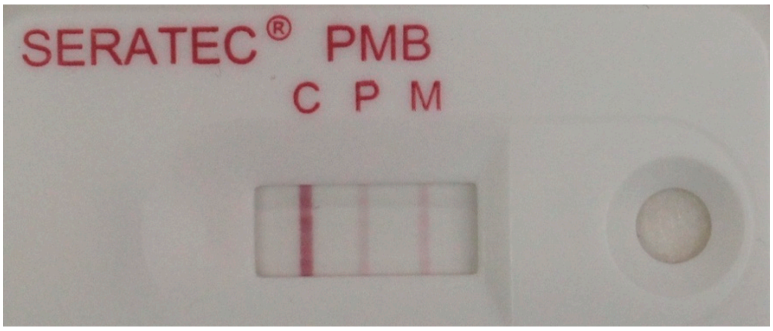

a) Hemoglobin and D-dimer bands: Strong

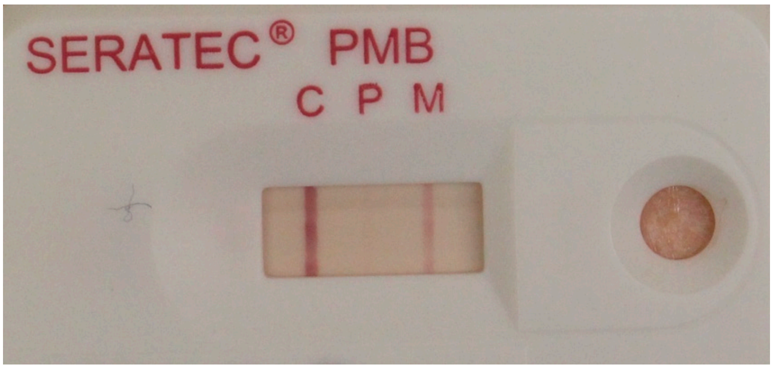

b) Hemoglobin band: Weak, D-dimer band: Strong

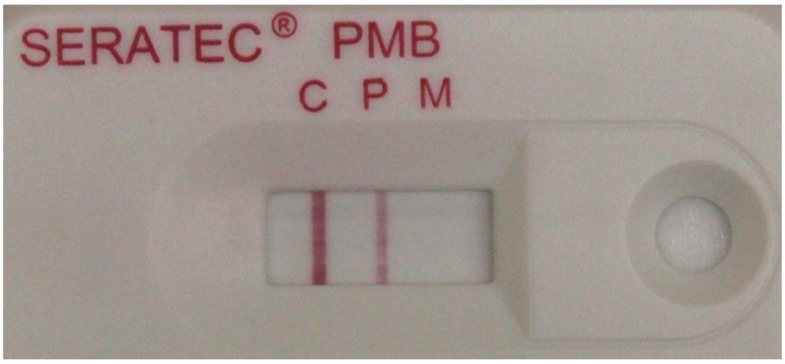

c) Hemoglobin band: Strong, D-dimer band: Negative

Figure S1. Examples of strong (a), weak (b), and negative (c) results

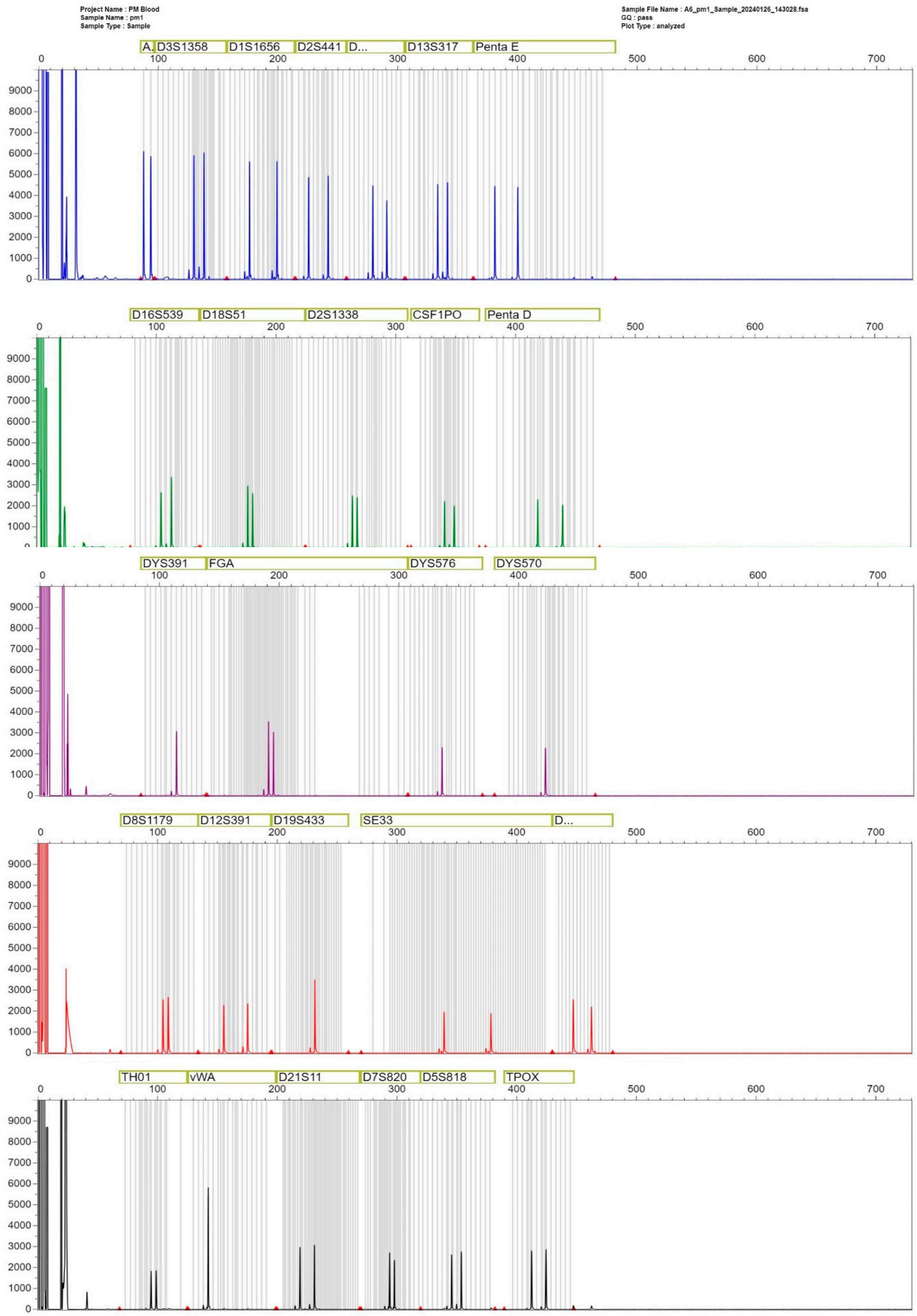

Figure S2. Full DNA profile from Postmortem Sample P001.
